# Batch-Dependent Hepatobiliary Toxicity of 10 nm Silver Nanoparticles After Single Intravenous Administration in Mice

**DOI:** 10.3390/nano16030176

**Published:** 2026-01-28

**Authors:** Simone Canesi, Laura Sala, Marcella de Maglie, Simona Rodighiero, Silvia Locarno, Andrea Raggi, Francesca Ferraris, Francesco Cubadda, Eugenio Scanziani, Cristina Lenardi, Camilla Recordati

**Affiliations:** 1 Department of Veterinary Medicine and Animal Sciences, University of Milan, Via dell’Università 6, 26900 Lodi, Italy; laura.sala2@unimi.it (L.S.); eugenio.scanziani@unimi.it (E.S.); 2 Mouse and Animal Pathology Laboratory, UNIMI Foundation, Viale Ortles 22/4, 20139 Milan, Italy; marcellademaglie@libero.it; 3 Department of Experimental Oncology, European Institute of Oncology (IEO IRCCS), Via Adamello 16, 20139 Milan, Italy; simona.rodighiero@ieo.it; 4 Department of Physics “Aldo Pontremoli”, University of Milan, Via Celoria 16, 20133 Milan, Italy; silvia.locarno@unimi.it (S.L.); cristina.lenardi@unimi.it (C.L.); 5 Istituto Nazionale di Fisica Nucleare (INFN), Sezione di Milano, Via Celoria 16, 20133 Milan, Italy; 6 Istituto Superiore di Sanità—National Institute of Health, Viale Regina Elena 299, 00161 Rome, Italy; andrea.raggi@iss.it (A.R.); francesca.ferraris@iss.it (F.F.); franscesco.cubadda@iss.it (F.C.)

**Keywords:** silver nanoparticles, intravenous administration, toxicity, batch-to-batch variability, in vivo study, mouse

## Abstract

Silver nanoparticles (AgNPs) are extensively employed for their antimicrobial and biomedical properties, yet concerns persist regarding their potential toxicity. While AgNPs can induce oxidative stress, membrane disruption, and DNA damage, in vivo data remain inconsistent. This study investigated whether batch-to-batch variability in nominally identical AgNPs of 10 nm size contributes to divergent in vivo toxicity outcomes. CD-1 (ICR) mice were intravenously injected with a single 10 mg/kg bw dose of spherical, citrate-coated 10 nm AgNPs from three different batches purchased from the same manufacturer. The mice were euthanized 24 h post-exposure for quantitative silver determination by inductively coupled plasma–mass spectrometry (ICP–MS) and histopathological evaluation of liver, spleen, lungs, kidneys, and brain. Autometallography and immunofluorescence were used to assess silver distribution and cellular localization in the hepatobiliary system. All the batches induced hepatobiliary toxicity, characterized by hepatocellular necrosis and gallbladder wall hemorrhage, of differing severity. The most toxic batches contained higher proportions of smaller AgNPs, suggesting that differences in size distribution influence toxicological outcomes. Silver agglomerates were localized within multiple cell types, indicating internalization and cell-specific cytotoxicity. These findings highlight that minor physicochemical variations affect in vivo results, underscoring the importance of nanoparticle characterization to improve reproducibility in nanotoxicological research.

## 1. Introduction

AgNPs are an important category of nanomaterials with high translational potential. Their small size (typically 1–100 nm) results in a high surface area to volume ratio, which is an important determinant of a spectrum of distinctive chemical, physical, and biological properties not shared by their bulk material counterparts [1].

AgNPs are used in several fields such as microelectronics, materials, textiles, energy, cosmetic goods, and healthcare [2]. AgNPs are renowned for the broad-spectrum and highly efficient antimicrobial properties against microorganisms, including bacteria, fungi, viruses, and parasites, and are also characterized by anticancer activity [3,4,5,6,7,8]. They are also used in wound repair, bone healing, as antidiabetic agents, in theranostics, as food contact materials, as adjuvants in dental materials, and in vaccines [9,10,11,12,13,14]. Considering the wide range of AgNPs applications in daily life, there is growing concern about aggregate human exposure by inhalation, dermal, oral, and parenteral routes, and their potential environmental impact [15].

Toxicological studies on AgNPs have been performed in vitro on bacteria and cell lines, and in vivo on a wide range of animal species, including mammals [16,17,18,19]. AgNPs can pass through biological membranes and enter cells, causing toxicity at different levels [20,21]. Distinct mechanisms of toxicity were reported at the cellular level, including membrane damage, reactive oxygen species (ROS) generation leading to oxidative stress, cytoskeleton and DNA degeneration, DNA repair enzymes damage, and autophagy upregulation [22,23,24]. These mechanisms can, in turn, lead to cytokine production, cellular damage, and eventually apoptosis and necrosis.

Compared to the consensus of in vitro studies, in vivo studies are controversial as to whether exposure to AgNPs causes adverse effects. Some studies indicate that AgNPs have toxic effects on different organs (lungs, liver, kidneys, spleen, brain, and parotid glands), either after single or repeated administration, and following different routes of exposure [25,26,27,28,29,30,31,32,33,34,35]. Other studies found no relevant adverse effects [36,37,38,39,40,41]. These contradictory results might depend on several factors, including the exposure experimental protocol, the experimental model, and the variable physicochemical properties of the tested AgNPs. In fact, AgNP toxicity is closely linked to shape, size, concentration, aggregation/agglomeration, chemical coating, surface charge, and process of synthesis [32,42,43,44]. Nevertheless, toxicological studies often consider these parameters as fixed within a given nanoparticle formulation, overlooking the possibility that subtle variations in one or more of these properties may occur between different production batches of nominally identical AgNPs.

The synthesis of nanoparticles relies on a variety of physical, chemical, and biological procedures [45]. Although in the production of AgNPs, and nanomaterials in general, the shape and size should be tightly controlled [45,46], current industrial and laboratory synthesis processes often result in significant batch-to-batch variability due to subtle variations in raw material purity, reaction conditions, and post-processing steps [47,48]. While such variability may be negligible for industrial performance, it can represent a major confounding factor in nanotoxicological studies, where relatively small differences within nominally identical batches may translate into inconsistent biological responses, ultimately undermining the reproducibility and comparability of toxicity studies [49,50,51]. Beer et al. reported a variation in toxicity between batches of AgNP suspensions in vitro, suggesting that these differences could be ascribed to some differences occurring in the physicochemical properties [52]. However, the direct impact of batch-to-batch variability on in vivo toxicological outcomes remains poorly investigated.

In view of the need to improve the understanding of the AgNP-related toxicity, this work aimed at investigating the impact of batch variability of 10 nm AgNPs on in vivo toxicity. Specifically, we sought to determine whether batch-to-batch variability within a single commercially available AgNP formulation—characterized by identical nominal size, coating, and supplier specifications—could result in distinct toxicological outcomes in vivo. The underlying hypothesis was that batch-to-batch variability could influence toxicological outcomes, potentially contributing to the inconsistent results on AgNP toxicity in the literature. Since this study was not intended to mimic human exposure scenarios, to avoid limited systemic exposure due to the cellular barriers present in the skin, gastrointestinal tract, and lungs, we used intravenous (IV) administration to evaluate the toxic effects induced by spherical 10 nm citrate (CT)-coated AgNPs. The selection of spherical 10 nm CT-coated AgNPs at a dose of 10 mg/kg was based on our earlier findings, where this size and dose combination showed measurable toxic effects, making it a relevant and sensitive model for assessing whether batch-to-batch differences can influence the toxicological response [32].

## 2. Materials and Methods

### 2.1. Physicochemical Characterization of Silver Nanoparticles

Suspensions of BioPure™ spherical Silver Nanoparticles of 5 nm (Batch no. MGM2185) and 10 nm (Batch A no. DAG1542, Batch B DAG1949, and Batch C DAG2289) in size, coated with CT, were purchased from NanoComposix (San Diego, CA, USA). All the suspensions were supplied at a concentration of about 1.0 mg/mL. BioPure™ AgNPs were chosen because they were guaranteed to be sterile and with an endotoxin level ≤ 5 EU/mL. For particle characterization, the CT-coated AgNPs were diluted to 2.0 mM in the suspending solvent, sodium citrate (cod. W302600, Sigma-Aldrich, Saint Louis, MO, USA) buffer. The samples were sonicated (Elmasonic S 30 H, Elma Schmidbauer GmbH, Singen, Germany) for 30 s, in accordance with the manufacturer’s instructions. To prevent contamination, measurements were performed using disposable plastic cuvettes. The AgNPs were tested immediately after their delivery, and in vivo experiments were run in the following week. Meanwhile, the AgNPs were stored at +4 °C, according to the manufacturer’s instructions. The quality of the dispersions was checked before the beginning of the in vivo trial by UV–visible (UV-vis) spectroscopy. Details provided by the manufacturer on the physicochemical properties of the investigated nanoparticles are included in Table S1.

The UV-vis spectra were acquired using a Cary 100 UV-vis spectrophotometer (Agilent Technologies, Santa Clara, CA, USA) in the wavelength range of 300–800 nm with steps of 1 nm. Due to the high UV-vis absorbance of the nanoparticles, AgNPs were diluted 1:200 in sodium citrate buffer (cod. W302600, Sigma-Aldrich, Saint Louis, MO, USA). All the measurements were run at room temperature at least three times on three different replicates.

### 2.2. Animals and Experimental Design

Male CD-1(ICR) mice of 4–5 weeks of age were purchased from Charles River (Calco, Italy). They were housed in standard Individually Ventilated Cages (IVC GM500, Tecniplast, Buguggiate, Italy) and acclimated to the environment for a week prior to the initiation of the study, with free access to water and standard pellet diet ad libitum. The environmental conditions were set at a temperature of 22 ± 2 °C, relative humidity of 55 ± 10%, and a 12 h light/dark cycle.

The mice were randomly assigned to five groups of treatments (n = 3) and IV-treated with a single injection into the lateral tail vein either with vehicle (sterile water) (control group) or 10 mg/kg body weight (bw) of 10 nm AgNPs of 3 different batches (Batch A no. DAG1542, Batch B no. DAG1949, and Batch C no. DAG2289) and 10 mg/kg bw of 5 nm AgNPs (Batch no. MGM2185) purchased from the same supplier (NanoComposix, San Diego, CA, USA). Immediately after the treatment and the following hours, the general health and behavior of the mice were monitored. The body weight of each mouse was measured before treatment and at sacrifice.

The mice were euthanized 24 h after IV administration by carbon dioxide inhalation using a gradual 20% vol/min displacement rate [53]. The animals were maintained according to the guidelines set out in Commission Recommendation 2007/526/EC and used in accordance with the Italian laws (D.L 26/2014) enforcing the Council Directive 2010/63/UE. The experiment was approved by the Italian Ministry of Health (approval no. 942/2015-PR, issued on 4 September 2015).

### 2.3. Sampling

A total of 24 h after IV administration, the mice were euthanized and underwent complete necropsy to collect the liver with gallbladder, spleen, kidneys, lungs, and brain for silver quantification and histopathological evaluation. The organ weight was measured, and relative organ weights (%) were calculated as wet organ weight/total body weight. For quantification of silver, a portion of the collected organs was stored at −80 °C for the later ICP-MS analysis. Unscheduled dead animals underwent the same procedure. In the mice treated with 5 nm AgNPs, organ weights were not recorded, and ICP-MS was not performed.

### 2.4. Quantification of Silver

The total Ag content was determined in the brain, spleen, kidney, lung, and liver by means of triple quadrupole ICP-MS. An 8800 ICPQQQ spectrometer (Agilent Technologies, Tokyo, Japan) equipped with an autosampler, a peristaltic pump, a Micro-Mist glass concentric nebuliser, and operated at a RF power of 1550 W, was used. All the sample manipulations were carried out in clean room conditions under a laminar flow box. The samples were placed in high-pressure Teflon containers with 3 mL of HNO_3_, 0.5 mL of H_2_O_2_ (both ultrapure grade, Carlo Erba, Rodano, Italy), and digested with a microwave system (UltraWAVE Single Reaction Chamber Microwave Digestion System, Milestone, Bergamo, Italy). The irradiation program consisted of 23 min at 220 °C (ramp), 10 min at 220 °C (hold, maximum power 1400 W), 15 min depressurization, and cooling at room temperature. After cooling, the digests were diluted by adding HCl (final concentration 3.0 M) to promote the formation of soluble silver complexes and prevent the precipitation of insoluble Ag^+^ salts. Prior to analysis the digests were highly diluted with 0.1% HNO_3_ and the appropriate amount of HCl to maintain silver in complexed form. Measurements were carried out on ^107^Ag and ^109^Ag by the method of external calibration using ^103^Rh as an internal standard. The method quantification limit ranged from 0.1 to 0.4 μg/kg tissue, depending on the tissue. Trueness was assessed by including in each analytical batch the certified reference material SRM 1577c Bovine Liver (NIST, Gaithersburg, MD, USA), with a certified Ag value of 5.9 ± 1.6 μg/kg and a found value of 6.0 ± 0.1 μg/kg.

### 2.5. Histology

For histological examination, the collected organs (hepatic median lobe including the gallbladder, spleen, kidneys, lungs, and brain) were fixed in 10% neutral buffered formalin for 48 h at room temperature, routinely processed for paraffin embedding, sectioned at 4 μm thickness, stained with Hematoxylin and Eosin (H&E), and evaluated under a light microscope. Grading of histopathological lesions in the examined organs was performed as follows: 0 = absence of lesions; 1 = minimal; 2 = mild; 3 = moderate; 4 = marked; 5 = severe.

To visualize the tissue distribution and cellular localization of silver, autometallography (AMG) staining [54,55] and immunofluorescence were performed on 4 μm serial sections. After AMG staining, sections were counterstained with safranin O and evaluated under a light microscope for the identification of tissue and cellular localization of silver, visible as black granular pigment. For immunofluorescence, liver sections were immunostained with the following primary antibodies: Iba-1 (macrophages) (Abcam, Cambridge, UK, clone EPR16589, rat monoclonal, ab283346, 1:1000), CD31 (endothelial cells) (Abcam, clone EPR17259, rabbit polyclonal, ab182981, 1:1000), Arginase-1 (hepatocytes) (Santa Cruz Biotechnology, Dallas, TX, USA, clone V20, goat polyclonal, sc-18354, 1:2500), and LYVE-1 (lymphatic endothelial cells) (Abcam, rabbit polyclonal, ab14917, 1:100). Sections underwent deparaffinization and heat induced epitope retrieval for 40 min at 96 °C (Dewax and HIER Buffer H, Thermo Scientific, Runcorn, UK, cat. no. TA-999-DHBH). Slides were incubated with PBS containing 10% normal serum for 30 min at room temperature to reduce nonspecific background staining and then incubated for 90 min at room temperature with primary antibodies. Sections were incubated with fluorescent secondary antibodies: Alexa Fluor^®^ 555 F(ab’)2 Fragment of donkey anti-rat, goat Anti-Rabbit IgG (H + L), and Alexa Fluor^®^ 488 F(ab’)2 Fragment of rabbit anti-goat (Life Technologies Europe BV, Monza, Italy, cat. No. A-21430, 1:200). Sections were counterstained with FluoroshieldTM with DAPI (Sigma-Aldrich, F6057). A known positive control section was included in each immunofluorescent-labeling assay. Images were acquired using the TCS SP8 confocal microscope (Leica Microsystems GmbH, Wetzlar, Germany) with a 63x/1.4 oil immersion objective, exciting and acquiring the emission of the Alexa 555 or Alexa 488 directly conjugated secondary antibodies, while nuclei were visualized by DAPI staining. Silver agglomerates were imaged in reflection mode using the 561 or 488 nm laser lines [37]. A Z-stack of a few microns was acquired for each field of view.

### 2.6. Statistical Analysis

Data were analyzed using GraphPad Prism version 10.0 (GraphPad Software, San Diego, CA, USA). Since the sample size (body and organ weights and silver concentrations) of in vivo experiments was small, a nonparametric test (Kruskal–Wallis test) was used to detect differences between groups. The *p*-values < 0.05 were considered statistically significant.

## 3. Results

### 3.1. Physicochemical Characterization of Silver Nanoparticles

Three commercial batches of CT-coated AgNPs with a nominal size of 10 nm and one batch of CT-coated 5 nm AgNPs purchased from the same supplier were tested in this study.

Before the investigation of their toxicological effects in vivo, physicochemical characterization was performed on purchased AgNPs to verify their conformity with the supplier’s specifications, as provided in the datasheets (Table S1). The results of UV-vis characterization compared with the values reported in their respective datasheets are summarized in Table 1, while full absorbance spectra of the tested silver nanoparticles are provided in Figure S1.

The characteristic maximum absorbance in the visible range, attributed to the surface plasmon resonance (SPR) effect [56], is closely related to AgNP morphology. Therefore, UV-vis spectroscopy provides valuable insights into variations in particle size and shape, and the presence of aggregates or agglomerates in the suspension. The measured values of λmax, representing the wavelength at which the maximum absorbance occurs, are consistent with those reported in the datasheet, with a variation between 0.7 and 1.3%. Similarly, the Hmax values, which indicate the amount of light absorbed at that specific wavelength, deviate by approximately 1% from the reference values. An exception is observed in the 10 nm Batch B, which showed a 9.6% variation in Hmax compared to the datasheet. Additionally, Dynamic Light Scattering (DLS) characterization of the hydrodynamic diameter in the liquid phase was performed, confirming negligible occurrence of either aggregates or agglomerates (Figure S2). DLS data are compared to the datasheet data in Table S2.

Given the agreement between the manufacturer’s specifications and our measurements, we ascertained the suitability of purchased AgNPs for in vivo experiments by ruling out the potential presence of aggregates that could affect the study outcomes.

**Figure 1:**
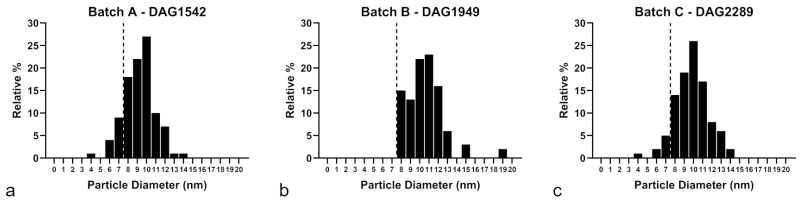
Number-based constituent particle size distribution reported in the manufacturer’s datasheets. (**a**) Batch A DAG1542; (**b**) Batch B, DAG1949; (**c**) Batch C DAG2289. Suspensions of BioPure™ 10 nm spherical Silver Nanoparticles, NanoComposix (San Diego, CA, USA). The AgNP suspensions exhibited distinct particle size distributions across the three batches: the proportion of particles with diameters < 8 nm was 14% in Batch A, 0% in Batch B, and 8% in Batch C. The dashed vertical line indicates the particle diameter threshold set at 8 nm.

Since the objective of this study was to investigate the batch-dependent toxicity of nominally identical 10 nm AgNPs, a more detailed analysis of the particle size distribution reported in the manufacturer’s datasheets was performed. Specifically, the three different 10 nm AgNP batches were compared to assess the proportion of particles with a diameter below 8 nm, revealing notable differences: Batch A contained approximately 14%, Batch B 0%, and Batch C 8% of particles with a diameter below 8 nm (Figure 1).

### 3.2. Animal Behavior, Mortality, and Weights

After administration of AgNPs and during the following 12 h, all mice appeared healthy, and no abnormal behavior was observed. However, 24 h after the treatment (on the next morning, when animals were examined before sacrifice), four mice (one mouse treated with Batch A of the 10 nm AgNPs, one treated with Batch C of the 10 nm AgNPs, and two mice treated with the 5 nm AgNPs) were found dead. At sacrifice, a reduction in body weight was observed in all the groups as compared to pre-treatment, with the most prominent reduction found in the mice treated with Batch A, although no significant differences in body weight gain were recorded (likely due to the small number of tested animals) (Table 2). No significant differences were observed in the relative and absolute organ weights between the treated and control mice (Table 2 and Table S3).

### 3.3. Pathology

Gross examination of the treated mice revealed the presence of circulatory changes in different locations, including hemorrhages, edema, and hyperemia, with variable incidence among the treatment groups (Table 3).

AgNP-related lesions were histologically observed in the examined organs (liver, gallbladder, spleen, kidneys, lungs, and brain) (Table 4).

**Figure 2:**
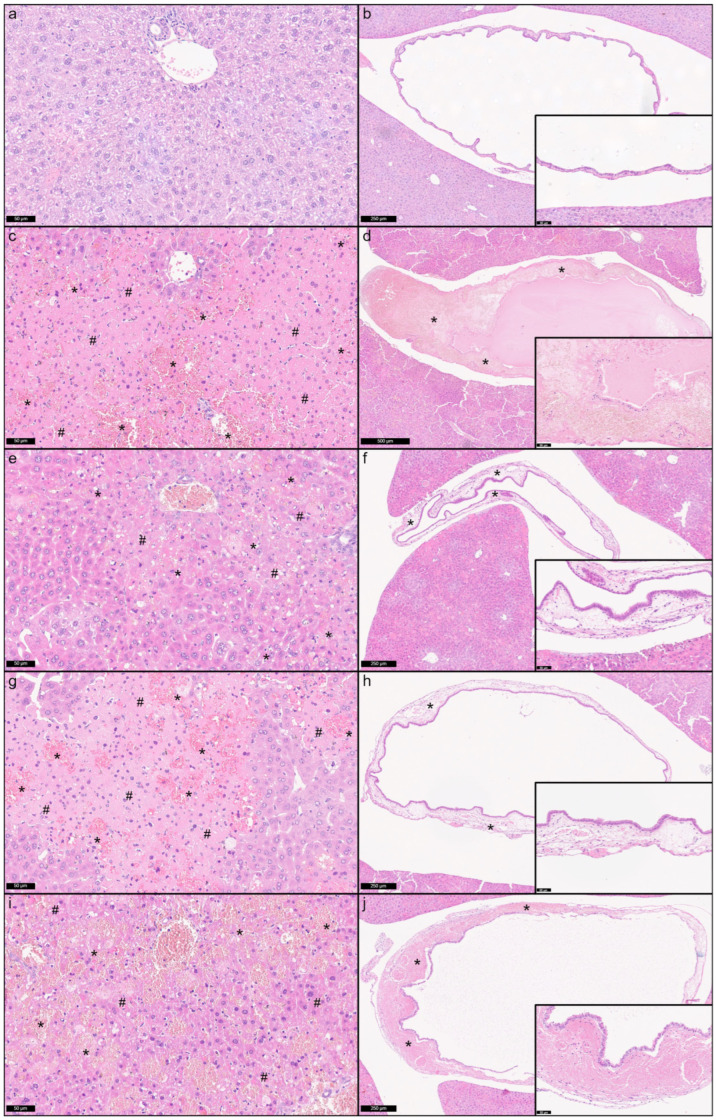
Hepatobiliary lesions induced by the three batches (Batch A, B, and C) of 10 nm AgNPs compared to 5 nm AgNPs 24 h after a single IV administration in mice: (**a**) vehicle, liver, H&E, 40×; (**b**) vehicle, gallbladder, H&E, 10×, inset 40×; (**c**) 10 nm AgNP Batch A, liver, H&E, 40×; (**d**) 10 nm AgNP Batch A, gallbladder, H&E, 7×, inset 40×; (**e**) 10 nm AgNP Batch B, liver, H&E, 40×; (**f**) 10 nm AgNP Batch B, gallbladder, H&E, 10×, inset 40×; (**g**) 10 nm AgNP Batch C, liver, H&E, 40×; (**h**) 10 nm AgNP Batch C, gallbladder, H&E, 10×, inset 40×; (**i**) 5 nm AgNPs, liver, H&E, 40×; (**j**) 5 nm AgNPs, gallbladder, H&E, 10×, inset 40×. No histological changes were present in the organs of the control mice. In the treated mice multifocal to diffuse periportal to midzonal hepatocellular necrosis and hemorrhages were present. Hemorrhages were more prevalent in Batch A-, Batch C-, and 5 nm treated animals. In the gallbladder, mural and intraluminal hemorrhages were also detected. Most severe lesions were observed in 10 nm Batch A and 5 nm. * = hemorrhage; # = necrosis.

All the 10 nm and 5 nm batches of AgNPs induced severe hepatobiliary lesions, including periportal to midzonal hepatocellular necrosis and hemorrhage, and mural and intraluminal hemorrhage of the gallbladder, while no lesions were present in the control mice injected with sterile water (Figure 2). These histopathological findings were most severe in the mice treated with the 5 nm AgNPs and more severe in the mice treated with Batches A and C compared to those treated with Batch B. In the Batch A-, C-, and 5 nm treated mice, hepatic hemorrhages were prevalent over necrosis, while in Batch B-treated mice, necrosis was the prevalent lesion. In the lungs, alveolar hemorrhages were observed only in Batch C and 5 nm, while variable degrees of hyperemia were found in the spleen, kidney, and lungs in all the examined groups. No intravascular silver agglomerates were observed in any of the examined organs of the treated mice, indicating the absence of thromboembolic lesions (Figure S3).

### 3.4. Tissue Distribution and Cellular Localization of Silver in the Hepatobiliary System

**Figure 3:**
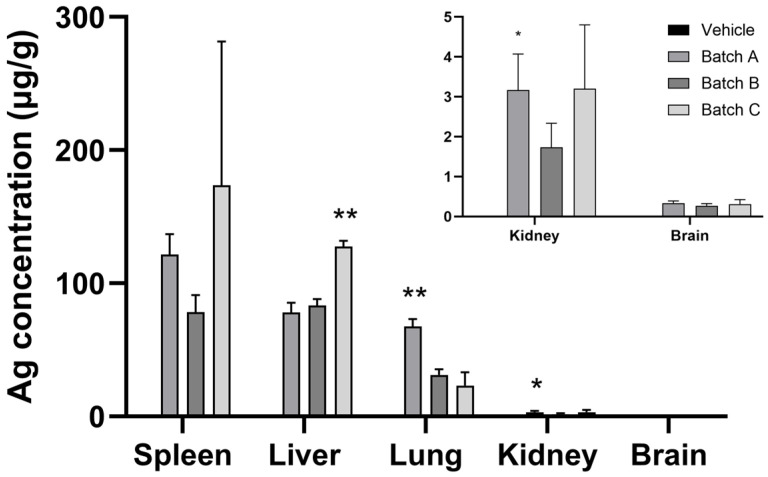
Silver tissue concentration 24 h after IV administration of vehicle or different batches of 10 nm AgNPs in mice. Data are expressed as mean ± SD. * *p* < 0.5; ** *p* < 0.01 vs. vehicle (Kruskal–Wallis test).

Distribution and localization of silver were evaluated in different organs 24 h after the IV administration of the CT-coated 10 nm AgNPs using two distinct but complementary approaches, i.e., ICP-MS and histologically, by using AMG staining. ICP-MS was used to quantitatively measure the total silver concentration in liver, spleen, kidneys, brain, and lungs, while AMG was used to histologically assess silver within the hepatobiliary system. The ICP-MS results indicated that silver concentration in the control group remained at background levels, whereas a marked increase was seen in key organs of the mononuclear phagocyte system. For all the 10 nm AgNP batches, the highest silver concentrations were found in the spleen and liver, followed by lungs, while in the kidney and brain, detected silver levels were orders of magnitude lower (Figure 3).

**Figure 4:**
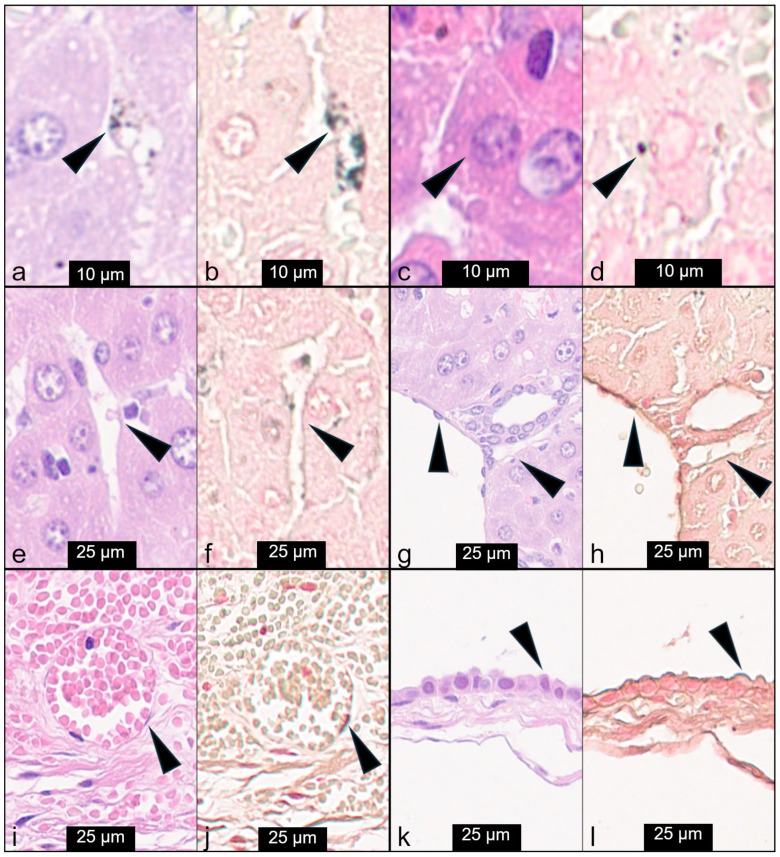
Detection of silver agglomerates in 10 nm CT-coated AgNP-treated mice: H&E and AMG staining performed on serial sections. (**a**) Kupffer cell, H&E; (**b**) Kupffer cell, AMG; (**c**) hepatocyte, H&E; (**d**) hepatocyte, AMG; (**e**) sinusoid, H&E; (**f**) sinusoid, AMG; (**g**) hepatic vascular and lymphatic endothelium, H&E; (**h**) hepatic vascular and lymphatic endothelium, AMG; (**i**) gallbladder vascular endothelium, H&E; (**j**) gallbladder vascular endothelium, AMG; (**k**) gallbladder epithelium, H&E; (**l**) gallbladder epithelium, AMG. Silver agglomerates, indicated by arrowheads, were clearly visible in Kupffer cells due to their phagocytic and accumulation activity, while in hepatocytes, endothelial cells, and gallbladder epithelium, the positivity was indicative but uncertain.

**Figure 5:**
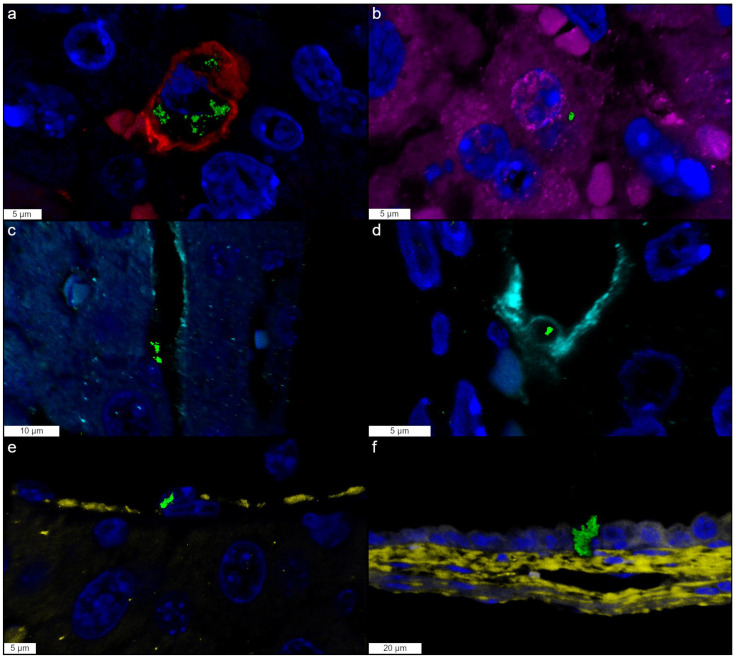
Localization of silver in liver and gallbladder evaluated by IF and confocal microscopy. Representative snapshots from the 3D rendering of the liver and gallbladder from 10 nm CT-coated AgNP-treated mice. Agglomerates of silver (green) are found within the cytoplasm of (**a**) Iba-1+ Kupffer cell (red); (**b**) Arginase-1+ hepatocytes (magenta); (**c**) LYVE-1+ lymphatic endothelium (light blue); (**d**) LYVE-1+ sinusoidal endothelium (light blue); (**e**) CD31+ vascular endothelial cell (yellow); (**f**) gallbladder epithelium. Nuclei are counterstained with DAPI (cyan).

In the H&E-stained sections, intracytoplasmic black granular pigment was barely visible in the liver in the centrilobular and necrotic areas, most likely in Kupffer cells, while no agglomerates were detected in the gallbladder. The hepatobiliary localization of silver was better detailed after AMG staining. In the liver most of the silver was mainly found in the cytoplasm of Kupffer cells, and rarely within hepatocytes. Silver was also suspected to be present in vascular, sinusoidal, and lymphatic endothelial cells, and within gallbladder epithelial cells (Figure 4). Through immunofluorescence and confocal microscopy, we were able to confirm the presence of silver agglomerates within all these cell types (Figure 5).

## 4. Discussion

In vivo studies on AgNPs have produced inconsistent toxicological outcomes, largely due to variability in their physicochemical properties and in experimental conditions. Even when nominally identical, AgNPs can differ in size distribution, surface chemistry, aggregation state, and synthesis-related features, factors known to influence biological behavior and toxicity. This variability is particularly relevant for biological and medical applications, where batch consistency is critical.

In this study, we examined whether such batch-to-batch differences among nominally 10 nm CT-coated AgNPs translate into divergent in vivo toxicity. The IV route of administration and the dose were selected based on our previous work, in which we observed hepatobiliary toxicity [32]. Although IV administration does not reflect a typical route of consumer exposure to AgNPs, it represents a relevant experimental approach for investigating systemic biodistribution and toxicity of AgNPs once they have crossed the primary biological barriers and entered the bloodstream, or when administered for clinical applications.

All the tested 10 nm AgNP batches induced hepatobiliary toxicity, confirming previous findings [32], although with variable incidence and severity. These findings indicate that nominally identical AgNPs can be associated with different toxicological effects, supporting the concept that batch-to-batch variability may contribute to divergent in vivo outcomes. Among the tested batches, Batch A, characterized by a higher proportion of particles smaller than 8 nm, caused the most severe effects in terms of mortality and hepatobiliary lesions, followed by Batch C and then Batch B (the one with the lowest proportion of smaller particles). Treatment with the 5 nm AgNPs, which was included specifically to verify whether the smallest particles accounted for the most severe toxic effects observed among the different 10 nm AgNP batches, resulted in high mortality (2 out of 3 mice were found dead) and severe gross and hepatobiliary lesions. The mortality observed in this study is considered a direct consequence of the acute toxicity of AgNPs, as complications related to the injection procedure or the formation of large agglomerates post-administration were reasonably excluded based on the delayed onset of mortality and histopathological findings (i.e., the absence of thromboembolic lesions associated with silver agglomerates, particularly in the lungs, which represent the first filter organ after IV administration).

Interestingly, the liver silver concentrations measured by ICP-MS were higher in the Batch C-treated mice than in the Batch A-treated mice, which is consistent with a larger proportion of smaller particles being deposited in this organ in the Batch A-treated mice. Since the mass of a spherical nanoparticle scales to the cube of its diameter, the higher tissue mass concentrations in the Batch C-treated mice are consistent with a greater deposition of larger particles compared to the Batch A-treated mice. On the other hand, silver tissue concentrations in the liver were lower for the Batch B-treated mice as compared to the Batch C-treated mice. Batch B showed a distinctive right-tailed size distribution with 11% of the constituent particles larger than 12 nm and sized up to 19 nm. There is substantial evidence supporting the existence of a size threshold around 10 nm, below which a dramatic increase in toxicity of AgNPs is seen [32,57,58]. This increased toxicity has been attributed to size-selective biological interactions, including enhanced cellular uptake of smaller AgNPs, although the extent of uptake may vary across different cell types (e.g., epithelial cells, endothelial cells, macrophages) [32]. Accordingly, the lower liver silver mass concentration in the Batch B-treated mice compared to the Batch C-treated mice may reflect limited internalization of the larger AgNPs (present only in Batch B), which, despite representing a small fraction of the particle number, contribute substantially to the total mass. Nevertheless, this interpretation remains speculative, as direct measurements of cellular uptake would have required mechanistically oriented in vitro experiments with appropriate cell models.

The liver and gallbladder were the target organs for the 10 nm and 5 nm AgNPs, likely due to the reported extensive biliary excretion of CT-coated AgNPs following IV administration [32,59]. Indeed, the cellular uptake of AgNPs and their interactions with cells and proteins are influenced by nanoparticle morphology. Spherical AgNPs demonstrate greater efficiency in traversing the vascular endothelium, interacting with endothelial cells, distributing within biological systems, and being internalized by cells compared to non-spherical nanoparticles [60,61]. Moreover, smaller AgNPs exhibit higher endocytic and exocytic efficiency and increased chemical activity, properties that have been associated with greater cytotoxicity potential [62,63]. Following internalization, AgNPs typically undergo lysosomal trafficking, where the acidic environment promotes their dissolution and the intracellular release of Ag^+^ ions, which are considered major drivers of AgNP-induced toxicity [15]. In summary, the available evidence indicates that smaller nanoparticles, due to their higher cellular penetration capacity and ion-mediated mechanisms, more readily enter and damage cells, resulting in more severe effects compared with larger particles.

A notable finding was the occurrence of circulatory changes (specifically hyperemia, edema, and hemorrhages) not only in the liver and gallbladder but also in other tissues, including the skin and subcutis, lymph nodes, thymus, lungs, spleen, intestine, and pancreas. These circulatory changes were prevalent in Batch A-, Batch C-, and in 5 nm treated mice, suggesting a potential acute cytotoxic effect of the smallest particles (<8 nm) on endothelial cells, although further studies are needed to provide direct mechanistic evidence of endothelial injury.

To explore potential cellular targets of 10 nm AgNPs, AMG staining and IF were performed in the liver and gallbladder. While H&E staining primarily revealed AgNPs within Kupffer cells as black agglomerates, AMG enhanced the detection of silver distribution in the liver and gallbladder across various locations, although it sometimes lacked clarity in identifying the specific cell types involved. To overcome this issue, IF was performed, and it enabled precise localization of silver within Kupffer cells, hepatocytes, endothelial cells (vascular, sinusoidal, and lymphatic), and biliary epithelial cells. This suggests that AgNPs can penetrate multiple cell types, leading to widespread cytotoxic effects. Their presence in the endothelium supports the hypothesis of direct endothelial cytotoxicity and its role in the onset of hemorrhagic lesions, while their localization within hepatocytes supports the hepatic cytotoxicity resulting in hepatocellular necrosis in the mice treated with 10 nm and 5 nm AgNPs.

The aim of this study was not to investigate size-dependent toxicity per se, but rather to address a critical and often overlooked issue in the nanotoxicology field: the reproducibility and translational reliability of in vivo studies. While batch variability and heterogeneity in particle size distributions have been discussed conceptually in the literature and demonstrated primarily in vitro, systematic investigations demonstrating the toxicological impact of such variability in vivo remain limited [51,52]. Specifically, this work provides direct in vivo evidence that batch-to-batch variability within a single commercially available AgNP formulation—nominally identical in primary size, coating, and supplier specifications—can lead to variable toxicological outcomes. This provides a plausible explanation for the inconsistencies in the results reported across in vivo studies and highlights an important limitation of relying solely on supplier specifications.

This study, however, has some limitations, mainly related to its exploratory nature. First, the small sample size (n = 3) and occurrence of mortality limited the statistical power of the analyses. Second, particle size distribution was assessed based on manufacturer-provided data rather than independent TEM characterization. Finally, other batch-to-batch differences (e.g., residual synthesis reagents and coating density) that could have contributed to batch variability were not investigated.

In conclusion, subtle differences among nominally identical AgNP batches can significantly influence in vivo outcomes. Our results demonstrate that the enhanced toxicity observed in some of the tested 10 nm AgNP batches was size-dependent, with the smallest particles inducing the most severe effects, particularly in terms of mortality and hepatobiliary injury. Therefore, comparisons of nanoparticle-induced toxicity should account for the physicochemical properties of the test materials to improve the reproducibility and reliable interpretation of in vivo toxicological studies.

## Figures and Tables

**Table 1:** Results of UV-vis characterization of the tested AgNPs compared with the values reported in their datasheets.

BioPure^TM^ Silver Nanoparticle	Lot n°	UV-Vis			
		λmax (nm)	λmax from Datasheet (nm)	Hmax (a.u.)	Hmax from Datasheet (a.u.)
10 nm Batch A	DAG1542	391	388	163.20	164.88
10 nm Batch B	DAG1949	392	389	145.60	161.05
10 nm Batch C	DAG2289	392	389	169.55	167.37
5 nm	MGM2185	401	396	131.30	132.85

**Table 2:** Body weight gain and relative organ weights at sacrifice (n = 3). Data are expressed as mean value ± SD.

Group	Body Weight Gain (%)	Liver (%)	Spleen (%)	Kidneys (%)	Lungs (%)	Brain (%)
Vehicle	−3.81 ± 4.49	6.86 ± 0.71	0.47 ± 0.1	1.74 ± 0.15	0.61 ± 0.05	1.71 ± 0.22
10 nm Batch A	−14.84 ± 0.83 *	7.26 ± 0.31	0.64 ± 0.04	1.68 ± 0.25	0.62 ± 0.10	1.75 ± 0.01
10 nm Batch B	−8.85 ± 3.91	6.13 ± 0.53	0.77 ± 0.07	1.62 ± 0.08	0.69 ± 0.07	1.73 ± 0.14
10 nm Batch C	−11.5 ± 4.84	7.13 ± 1.23	0.55 ± 0.08	1.80 ± 0.13	1.55 ± 0.86	1.70 ± 0.63
5 nm	−6.67 ± 4.84	n.a.	n.a.	n.a.	n.a.	n.a.

* Body weight of the dead animal was not available (n.a.).

**Table 3:** Incidence of gross findings induced after a single IV injection of 10 and 5 nm AgNPs (acute toxicity).

Organ	Gross Finding	Vehicle	10 nm Batch A	10 nm Batch B	10 nm Batch C	5 nm
External examination	Epistaxis	0/3	0/3	0/3	1/3	2/3
	Digits, hyperemia	0/3	1/3	0/3	1/3	2/3
Subcutis	Inguinal lymph nodes, red discoloration *	0/3	1/3	0/3	0/3	1/3
	Red discoloration *	0/3	1/3	0/3	0/3	0/3
Abdominal cavity	Peritoneal effusion	0/3	1/3	0/3	0/3	0/3
Liver	Enlargement (hyperemia)	0/3	1/3	1/3	0/3	2/3
Gallbladder	Red discoloration *	0/3	0/3	0/3	1/3	2/3
Spleen	Enlargement (hyperemia)	0/3	2/3	2/3	2/3	2/3
Intestine	Red discoloration *	0/3	1/3	0/3	0/3	1/3
	Edema	0/3	0/3	0/3	0/3	1/3
Mesenteric lymph nodes	Red discoloration *	0/3	1/3	0/3	0/3	0/3
Pancreas	Edema	0/3	0/3	0/3	0/3	1/3
Pancreatic lymph nodes	Red discoloration *	0/3	2/3	0/3	0/3	0/3
Lungs	Red discoloration *	0/3	1/3	2/3	0/3	0/3
Thymus	Red discoloration *	0/3	1/3	0/3	0/3	0/3
Brain	Leptomeninges, hyperemia	0/3	1/3	0/3	0/3	0/3

* Not possible to discriminate between hyperemia, hemorrhage, or both.

**Table 4:** Incidence and median (range) severity score of histological findings induced after 24 h after a single IV injection of 10 and 5 nm AgNPs (acute toxicity). The following grading system was applied: 0 = absence of lesions; 1 = minimal; 2 = mild; 3 = moderate; 4 = marked; 5 = severe.

Organ	Histological Finding		Vehicle	10 nm Batch A	10 nm Batch B	10 nm Batch C	5 nm
Liver	Necrosis and/or hemorrhage	Incidence	0/3	3/3	3/3	3/3	3/3
		Median (range)	0 (0–0)	5 (4–5)	3 (1–4)	5 (4–5)	5 (4–5)
Gallbladder	Hemorrhage	Incidence	0/3	3/3	2/3	1/2 *	3/3
		Median (range)	0 (0–0)	2 (1–5)	2 (0–2)	1 (0–2)	4 (3–4)
Spleen	Red pulp, hyperemia	Incidence	0/3	2/3	2/3	1/3	3/3
		Median (range)	0 (0–0)	2 (0–4)	2 (0–4)	0 (0–2)	4 (3–5)
	White pulp, lymphocyte apoptosis	Incidence	0/3	1/3	0/3	0/3	2/3
		Median (range)	0 (0–0)	0 (0–3)	0 (0–0)	0 (0–0)	2 (0–2)
Kidney	Hyperemia	Incidence	0/3	1/3	0/3	1/3	2/3
		Median (range)	0 (0–0)	0 (0–2)	0 (0–0)	0 (0–2)	0 (0–2)
Lung	Hyperemia	Incidence	0/3	1/3	1/3	2/3	3/3
		Median (range)	0 (0–0)	0 (0–3)	2 (0–2)	0 (0–3)	3 (2–4)
	Alveolar hemorrhage	Incidence	0/3	0/3	0/3	1/3	2/3
		Median (range)	0 (0–0)	0 (0–0)	0 (0–0)	0 (0–1)	1 (0–2)

* The gallbladder of the dead mouse was not evaluated due to postmortem changes.

## Data Availability

The original contributions presented in this study are included in the article/Supplementary Material. Further inquiries can be directed to the corresponding author.

## Supplementary Materials

The following supporting information can be downloaded at https://www.mdpi.com/article/10.3390/nano16030176/s1. Table S1: Physicochemical properties of the investigated silver nanoparticles provided by the manufacturer; Figure S1: Particle characterization by UV–vis spectroscopy: full absorbance spectra of the tested silver nanoparticles; Table S2: AgNP hydrodynamic diameter by Dynamic Light Scattering comparison between analysis performed in the present study and data provided by the manufacturer; Figure S2: AgNP hydrodynamic diameter by Dynamic Light Scattering; Table S3: Absolute body and organ weights; Figure S3: Representative histological images of lung tissue from mice intravenously exposed to different batches of 10 nm and 5 nm AgNPs.

## Abbreviations

The following abbreviations are used in this manuscript:

AgNPs	Silver Nanoparticles
ICP–MS	Inductively Coupled Plasma–Mass Spectrometry
ROS	Reactive Oxygen Species
NPs	Nanoparticles
CT	Citrate
UV-vis	UV–Visible Spectroscopy
DLS	Dynamic Light Scattering
IV	Intravenous
IVC	Individually Ventilated Cage
bw	Body Weight
H&E	Hematoxylin and Eosin
AMG	Autometallography Staining
