# Supplementary material for: Batch-Dependent Hepatobiliary Toxicity of 10 nm Silver Nanoparticles After Single Intravenous Administration in Mice
Source: Nanomaterials (Basel). 2026 Jan 28;16(3):176. doi: 10.3390/nano16030176 (PMC12899605; doi:10.3390/nano16030176)
Supplement: Supplementary file 1 [file nanomaterials-16-00176-s001.zip › nanomaterials-4069149-supplementary.pdf]

nanomaterials

Article

Batch-Dependent Hepatobiliary Toxicity of 10 nm Silver Nanoparticles After Single Intravenous Administration in Mice

Simone Canesi [1,2,*], Laura Sala [1,2], Marcella de Maglie [2], Simona Rodighiero [3], Silvia Locarno [4,5], Andrea Raggi [6], Francesca Ferraris [6], Francesco Cubadda [6], Eugenio Scanziani [1,2], Cristina Lenardi [4,5] and Camilla Recordati [1,2,*]

[1] Department of Veterinary Medicine and Animal Sciences, University of Milan, Via dell'Università 6, 26900 Lodi, Italy; laura.sala2@unimi.it (L.S.); eugenio.scanziani@unimi.it (E.S.)
[2] Mouse and Animal Pathology Laboratory, UNIMI Foundation, Viale Ortles 22/4, 20139 Milan, Italy; marcellademaglie@libero.it
[3] Department of Experimental Oncology, European Institute of Oncology (IEO IRCCS), Via Adamello 16, 20139 Milan, Italy; simona.rodighiero@ieo.it
[4] Department of Physics "Aldo Pontremoli", University of Milan, Via Celoria 16, 20133 Milan, Italy; silvia.locarno@unimi.it (S.L.); cristina.lenardi@unimi.it (C.L.)
[5] Istituto Nazionale di Fisica Nucleare (INFN), Sezione di Milano, Via Celoria 16, 20133 Milan, Italy
[6] Istituto Superiore di Sanità—National Institute of Health, Viale Regina Elena 299, 00161 Rome, Italy; andrea.raggi@iss.it (A.R.); francesca.ferraris@iss.it (F.F.); franscesco.cubadda@iss.it (F.C.)
* Correspondence: simone.canesi@unimi.it (S.C.); camilla.recordati@unimi.it (C.R.)

Supplementary Materials

Table S1. Physicochemical properties of the investigated silver nanoparticles provided by the manufacturer.

BioPure™ Silver nanoparticle	Lot n°	Batch	DLS Mean hydrodynamic diameter (nm)	UV-vis Λmax (nm)	UV-vis Hmax (a.u)	TEM Ferret diameter (mean ± SD) (nm)	TEM Ferret diameter D90* (nm)	Mass conc. (mg/ml)	Particle conc. (n°/ml)	Solvent (mM)	pH
10 nm	DAG1542	A	n.a.	388	164.88	8.8 ± 1.7	11.0	1.03	3.5 x 10^{14}	Citrate 2.0	7.3
10 nm	DAG1949	B	15.4	389	161.05	10.0 ± 1.8	13.0	1.00	1.8 x 10^{14}	Citrate 2.0	7.6
10 nm	DAG2289	C	14.5	389	167.37	9.4 ± 1.7	12.0	1.07	2.4 x 10^{14}	Citrate 2.0	7.7
5 nm	MGM2185	D	n.a.	396	132.85	5.1 ± 0.7	n.a.	1.09	1.5 x 10^{15}	Citrate 2.0	7.8

n.a. = not available; * = 90th percentile of the constitutent PSD.

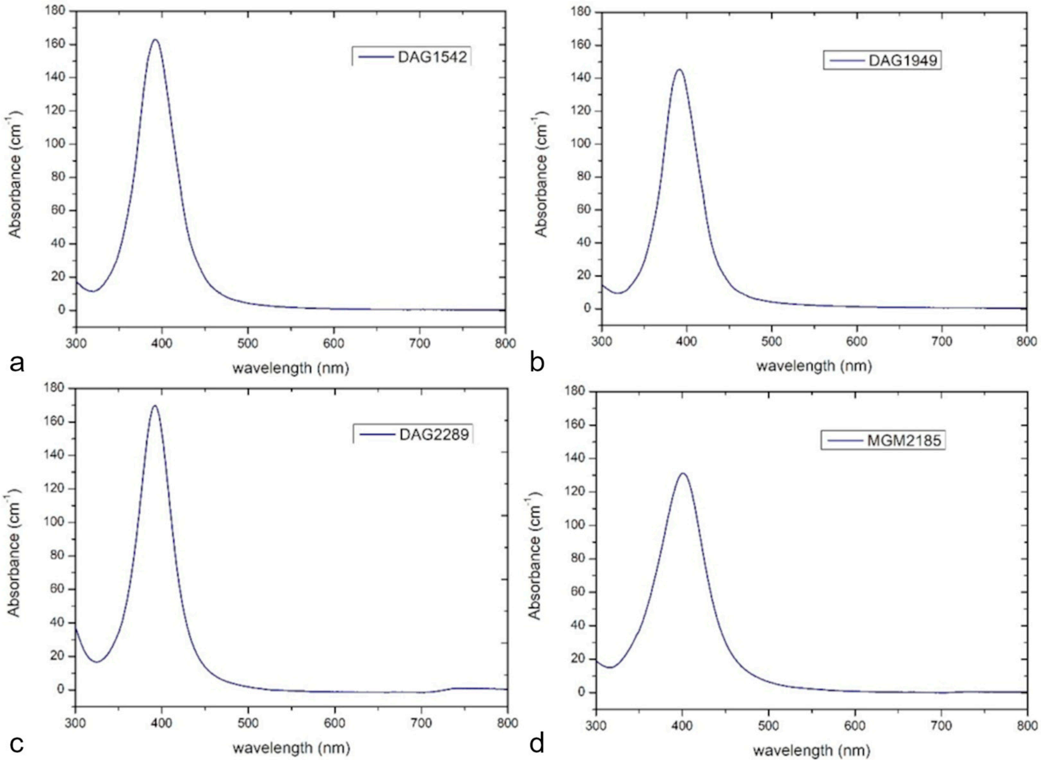

Figure S1. Particle characterization by UV–vis spectroscopy: full absorbance spectra of the tested silver nanoparticles. (**a**) 10 nm AgNPs Batch A, DAG 1542; (**b**) 10 nm AgNPs Batch B, DAG1949; (**c**) 10 nm AgNPs Batch C, DAG2289; (**d**) 5 nm AgNPs, MGM2185. The optical density in the 600-800 nm range, which is typical for aggregate absorption, was not detected in any of tested AgNPs, indicating the absence of stable aggregates in these samples. Hmax and λmax obtained from this analysis were compared to the values reported by the manufacturer's datasheets.

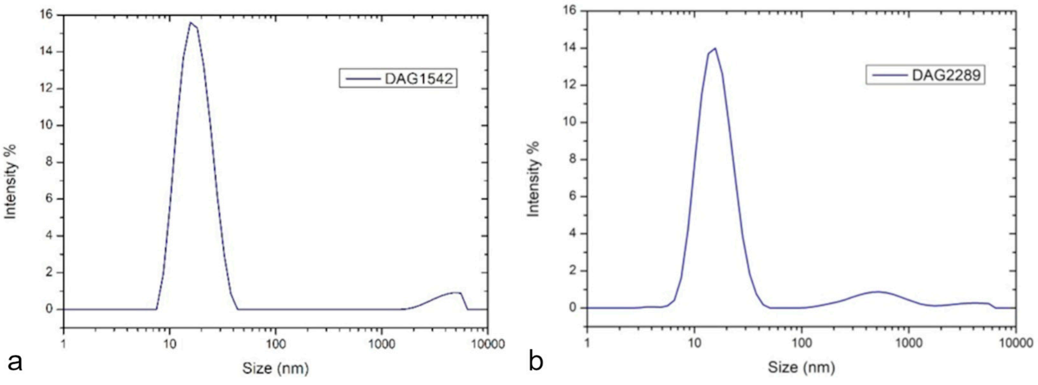

Figure S2. AgNPs hydrodynamic diameter by Dynamic Light Scattering. (**a**) 10 nm AgNPs Batch A, DAG 1542; (**b**) 10 nm AgNPs Batch C, DAG2289. The actual size, referred to hydrodynamic diameter, of AgNPs in dispersion was measured by DLS. Size distribution measurements were carried out in low volume disposable cuvettes using a Malvern Zetasizer Nano ZS90 instrument at 25 °C, equipped with 633 nm solid state He-Ne laser at a scattering angle of 90°. Analyses were performed in aqueous 2.0 mM sodium citrate buffer (cod. W302600, Sigma-Aldrich). The size measurements were averaged from at least three repeated measurements.

Table S2. AgNPs hydrodynamic diameter by Dynamic Light Scattering comparison between analysis performed in the present study and data provided by the manufacturer.

BioPure™ Silver	Lot n°	Batch	DLS

**nanoparticle**			**Mean hydrodynamic diameter** (nm)	**Mean hydrodynamic diameter from datasheet** (nm)
10 nm	DAG1542	A	18.0	n.a.
10 nm	DAG1949	B	n.a.	15.4
10 nm	DAG2289	C	16.5	14.5
5 nm	MGM2185	D	n.a.	n.a.

n.a. = not assessed.

Table S3. Absolute body and organ weights.

Group	Body weight (g)	Body weight gain (g)	Liver (g)	Spleen (g)	Kidneys (g)	Lungs (g)	Brain (g)
Vehicle	27.87 ± 2.73	-1.06 ± 1.21	1.92 ± 0.36	0.13 ± 0.06	0.49 ± 0.08	0.17 ± 0.02	0.47 ± 0.02
10 nm Batch A	24.34 ± 0.56	-4.25 ± 0.37	1.85 ± 0.16	0.15 ± 0.01	0.41 ± 0.05	0.17 ± 0.05	0.45 ± 0.04
10 nm Batch B	27.68 ± 1.90	-2.67 ± 1.11	1.69 ± 0.08	0.21 ± 0.03	0.45 ± 0.04	0.19 ± 0.02	0.48 ± 0.01
10 nm Batch C	24.99 ± 1.30	-3.28 ± 1.43	1.89 ± 0.29	0.15 ± 0.03	0.48 ± 0.07	0.42 ± 0.26	0.45 ± 0.17
5 nm	28.58 ± 0.26	-2.08 ± 0.67	n.a	n.a	n.a	n.a	n.a

Body weight of the dead animal was not available (n.a.).

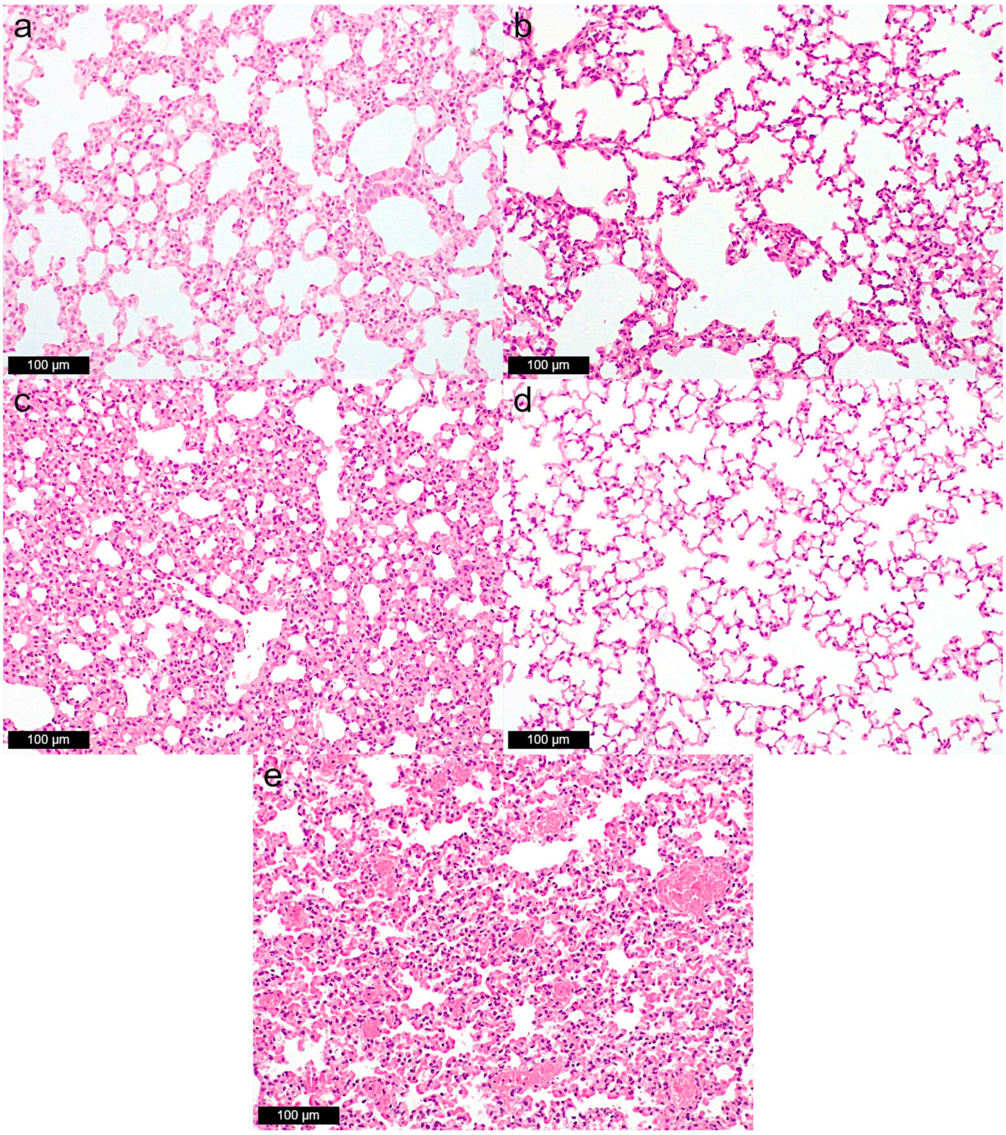

Figure S3. Representative histological images of lung tissue from mice intravenously exposed to different batches of 10 nm and 5 nm AgNPs. (**a**) Vehicle, H&E, 20x; (**b**) 10 nm AgNPs Batch A, H&E, 20x; (**c**) 10 nm AgNPs Batch B, H&E, 20x; (**d**) 10 nm AgNPs Batch C, H&E, 20x; (**e**) 5 nm AgNPs, H&E, 20x. Across all treatment groups no evidence of intravascular silver agglomerates or thromboembolic occlusive lesions were observed.

https://doi.org/10.3390/nano16030176
