# Supplementary figures and images for: Batch-Dependent Hepatobiliary Toxicity of 10 nm Silver Nanoparticles After Single Intravenous Administration in Mice
Source: Nanomaterials (Basel). 2026 Jan 28;16(3):176. doi: 10.3390/nano16030176 (PMC12899605; doi:10.3390/nano16030176)

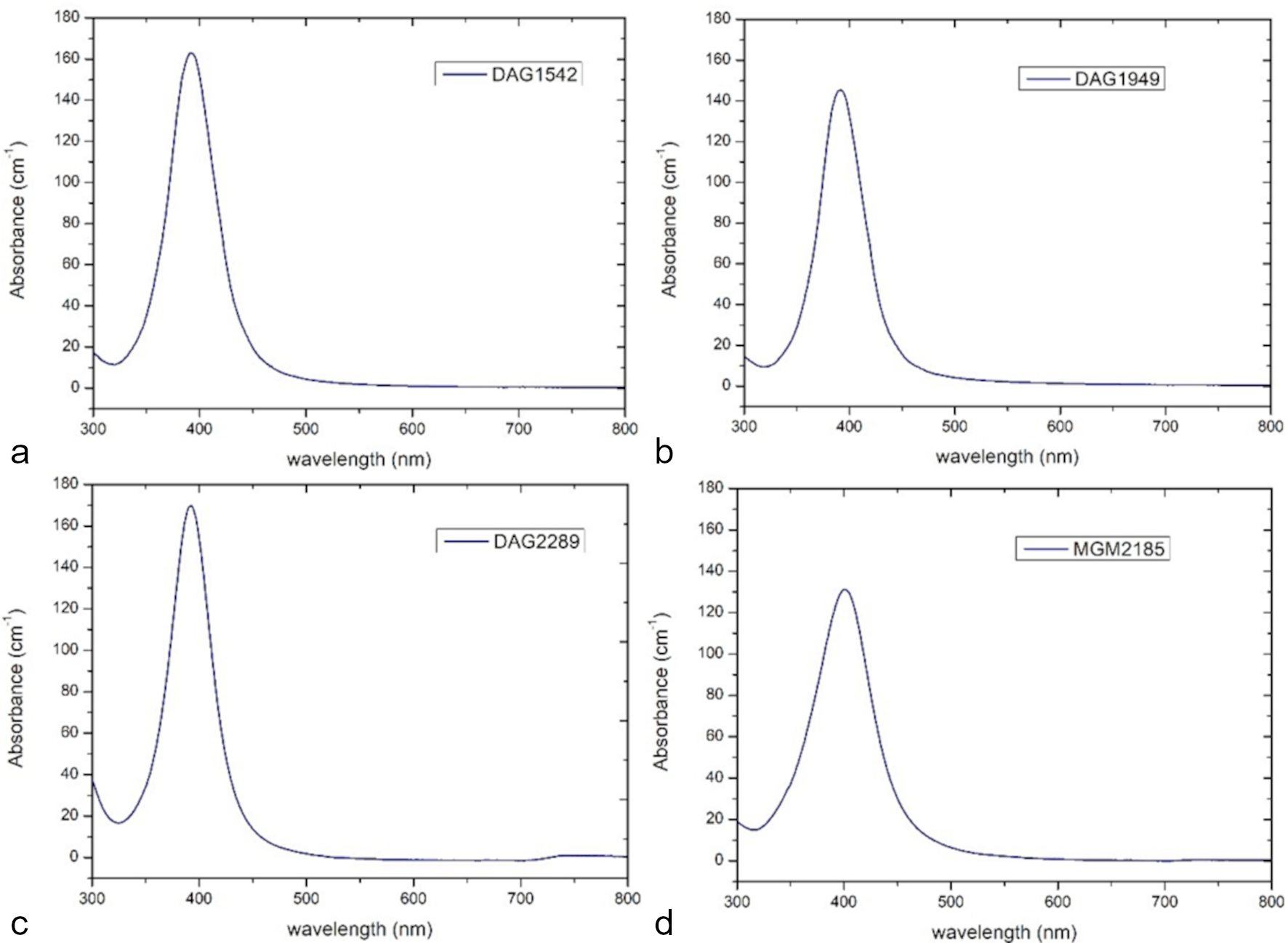

Supplement: Supplementary file 1 [file nanomaterials-16-00176-s001.zip › Supplemental figure 1.tif]

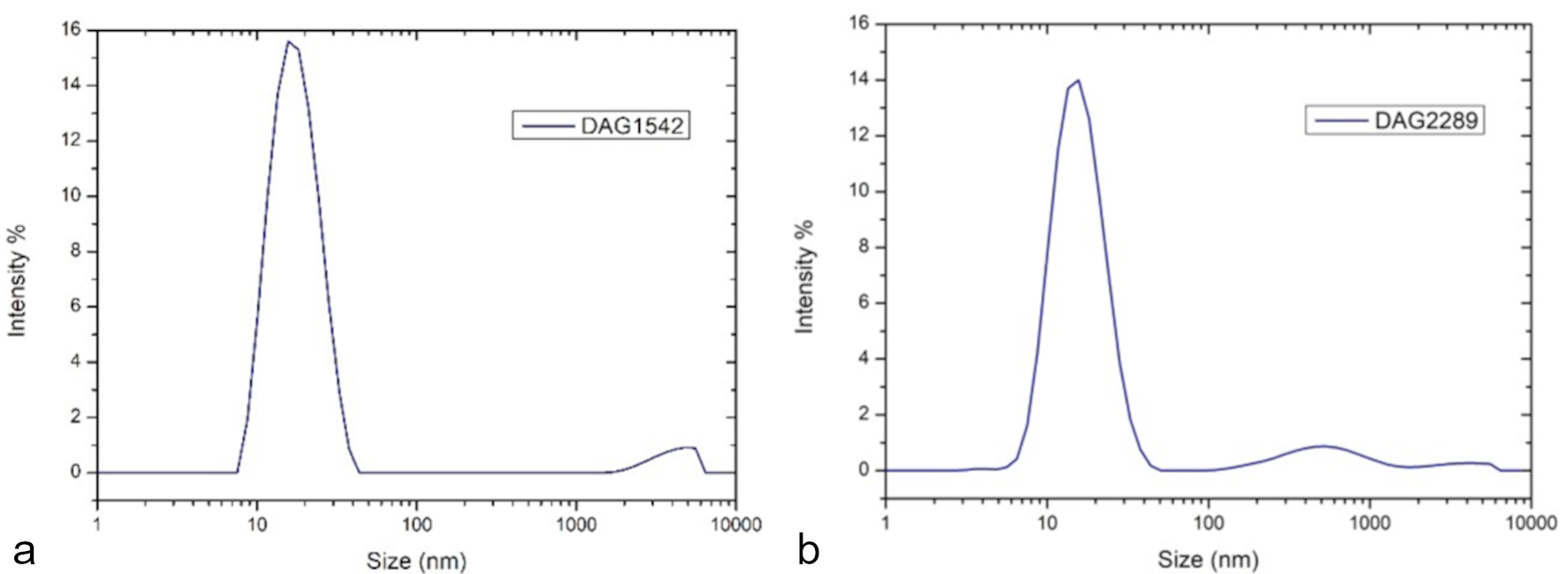

Supplement: Supplementary file 1 [file nanomaterials-16-00176-s001.zip › Supplemental figure 2.tif]

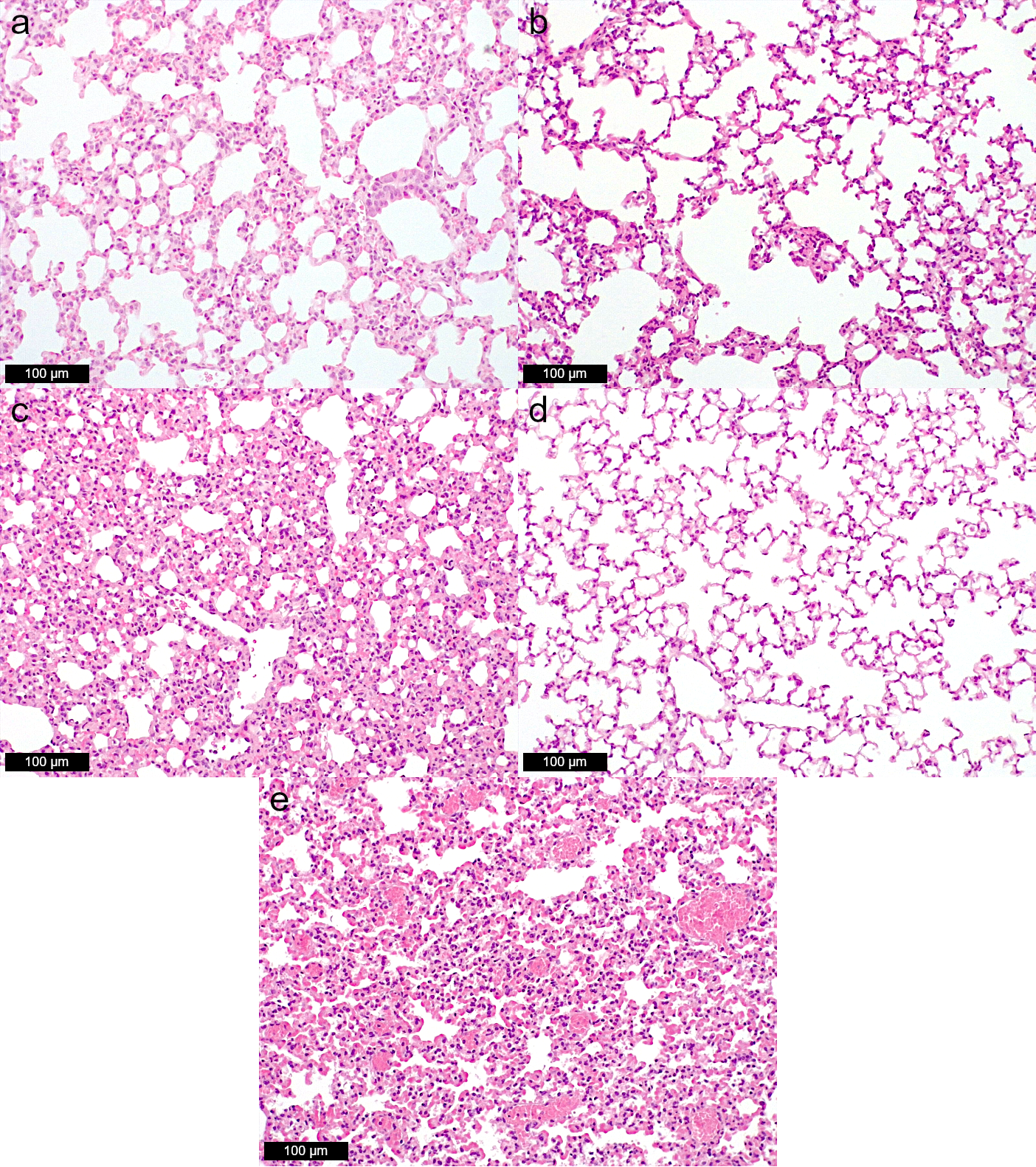

Supplement: Supplementary file 1 [file nanomaterials-16-00176-s001.zip › Supplemental Figure 3.tif]
